# The non-linear relationship between the visceral adiposity index and the risk of prediabetes and diabetes

**DOI:** 10.3389/fendo.2025.1407873

**Published:** 2025-03-21

**Authors:** Lan Huang, Jing Liao, Chunyan Lu, Yiqiong Yin, Yanling Ma, Yue Wen

**Affiliations:** Division of Gastrointestinal Surgery, Department of General Surgery, West China Hospital, Sichuan University/West China School of Nursing, Sichuan University, Chengdu, China

**Keywords:** visceral adiposity index, prediabetes, diabetes, non-linear relationship, NHANES

## Abstract

**Background:**

The visceral adiposity index is a valuable tool for assessing visceral fat accumulation. However, its non-linear association with prediabetes and diabetes requires further elucidation. Therefore, we aim to clarify the intricate interplay between the visceral adiposity index and these dysglycemic conditions.

**Methods:**

The National Health and Nutrition Examination Survey database from 1999 to 2018 was utilized to analyze health data from 24,072 participants. A multivariate logistic regression model was employed to evaluate the independent association between the visceral adiposity index and prediabetes and diabetes while considering potential confounding factors. Generalized additive models were used to identify any non-linear relationships by fitting smooth curves. Additionally, a stratified analysis based on different baseline characteristics was conducted, along with an interactive analysis.

**Results:**

After accounting for all relevant variables, individuals in the lowest quartile of the visceral adiposity index had a notably diminished likelihood of progressing to prediabetes and diabetes when compared with those in the other three quartiles. The odds ratios and 95% confidence intervals were as follows: 1.37 (1.23, 1.53), 1.87 (1.65, 2.12), and 2.80 (2.33, 3.37). More importantly, a non-linear association was observed between the visceral adiposity index and prediabetes and diabetes, with a threshold identified at 2.10.

**Conclusions:**

There exists a notable and positive association between the visceral adiposity index and prediabetes and diabetes, displaying non-linear attributes in this evaluation of the relationship. Risk assessment and early prevention strategies targeting the maintenance of low levels of visceral adiposity index may substantially diminish the likelihood of developing prediabetes and diabetes.

## 1 Introduction

The incidence of diabetes, a chronic metabolic disease characterized by hyperglycemia, has been rising in recent decades. Moreover, it has become one of the principal contributors to global mortality and disability, imposing significant medical and economic burdens (1). In 2021 alone, approximately 529 million individuals were affected by diabetes globally, with projections indicating a staggering increase to 1,310 million by 2050 (2). The prediabetic condition signifies a substantial risk for progressing to diabetes, and its importance in public health must not be overlooked (3). Projections indicate that more than 470 million people are expected to be affected by prediabetes by 2030, exhibiting twice the incidence rate compared to diabetes itself (4).

Furthermore, an alarming statistic revealed that approximately 5% to 10% of those with prediabetes will progress to full-blown diabetes annually. This figure escalates to an astonishing 50% after 10 years (4, 5). A strong association was found between prediabetes and heightened risks for stroke, cardiovascular disease, kidney disease, and all-cause mortality (6–9). Additionally, several investigations have shown that individuals in the prediabetic state have the opportunity to reverse the condition and return to normal glucose metabolism before developing diabetes (10). Therefore, efficient management strategies necessitate the early detection of prediabetes along with individualized interventions aimed at reducing the burden imposed by diabetes while preventing associated complications.

Obesity, particularly the accumulation of visceral fat, has been firmly linked to a broad spectrum of metabolic disorders (11). Visceral fat accumulation can stimulate excessive secretion of pro-inflammatory factors and adipokines, exacerbating insulin resistance and abnormal blood glucose levels (12). While magnetic resonance imaging and computed tomography (CT) are the best methods for measuring visceral fat, their application in large-scale population screening is limited by cost, complexity, and potential radiation exposure (13). In 2010, Amato et al. introduced the visceral adiposity index (VAI), a novel way to quantify visceral adiposity using waist circumference (WC), body mass index (BMI), triglyceride (TG) levels, and high-density lipoprotein cholesterol (HDL-C) (14). Studies have shown a strong concordance between VAI and CT measurement of visceral fat, which can better predict the occurrence of glucose and lipid metabolism disorders. Furthermore, VAI exhibited an inverse correlation with insulin sensitivity (14). Compared to conventional indicators of adiposity, VAI exhibits superior predictive performance across diverse populations with chronic diseases, serving as a simple and efficacious tool for assessing visceral fat accumulation and dysfunction (15–17). Additionally, an association between VAI and diabetes has been reported, with VAI potentially acting as an independent predictor of diabetes (18).

However, limited research has considered the diabetic population collectively, leaving the complex associations between VAI and prediabetes and diabetes largely unexplored. Furthermore, few previous studies have involved U.S. populations. Therefore, our objective was to evaluate the connection between VAI and prediabetes and diabetes by analyzing data from the National Health and Nutrition Examination Survey (NHANES), while also exploring any potential non-linear associations.

## 2 Methods

### 2.1 Study population

Data from the NHANES, a nationally representative survey of American civilians, was used in this study. The database employs a comprehensive multi-stage complex sampling methodology and incorporates data obtained from questionnaires, physical examinations, and laboratory tests, all of which are publicly accessible. All the participants signed an informed consent form. Furthermore, the study protocol received prior approval from the Institutional Review Board of the National Center for Health Statistics (NCHS).

**Figure 1:**
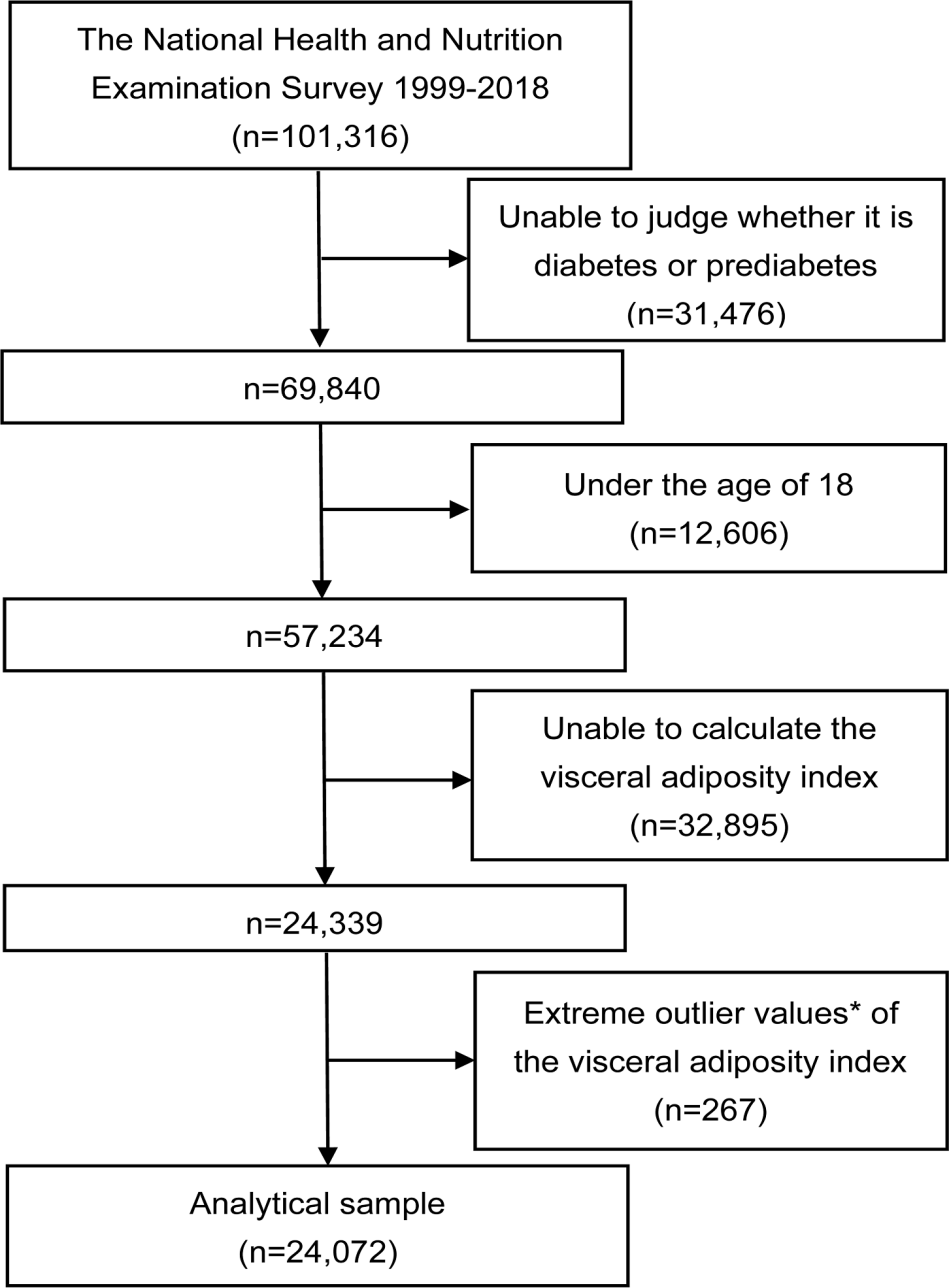
Flowchart of the study. ^*^Extreme outlier values were defined as those over 3 standard deviations from the mean.

The study was a cross-sectional study that utilized NHANES data spanning from 1999 to 2018, encompassing a total of 101,316 initial participants across the 10 consecutive survey cycles. Exclusion criteria were patients who did not have a determined prediabetes and diabetes status (n=31,476), who were younger than 18 years (n=12,606), who did not have a calculated VAI (n=32,895), and who had an extreme VAI value (mean ± 3 standard deviations) (n=267). Finally, a total of 24,072 eligible participants were included in the analyses (**Figure 1**).

### 2.2 Exposure and outcome variables

The exposure variable in this study was VAI, a trustworthy tool for evaluating visceral fat function (14). The computation formula for VAI is as follows: for men, VAI = [WC (cm)/(39.68 + 1.88 x BMI (kg/m2))] × (TG (mmol/L)/1.03) × (1.31/HDL-C (mmol/L)]; for women, VAI = [WC (cm)/(36.58 + 1.89 x BMI (kg/m2))]×(TG(mmol/L)/0.81)×(1.52/HDL-C (mmol/L)).

Prediabetes and diabetes were included as outcome variables in our study. Prediabetes was determined according to any of the following criteria: diagnosed by a physician or health professional, fasting plasma glucose (FPG) levels ranging from 5.6 to 7 mmol/L, glycosylated hemoglobin (HbA1c) levels ranging from 5.7% to 6.5%, or an FPG value during a 2-hour oral glucose tolerance test (OGTT) ranging from 7.8 mmol/L to 11.0 mmol/L. Diabetes was defined as a self-reported physician diagnosis, HBA1c level greater than 6.5%, FPG level greater than 7 mmol/L, or 2-hour OGTT plasma glucose level greater than 11.1 mmol/L. In this study, combined prediabetes and diabetes were analyzed as an outcome variable.

### 2.3 Covariates

Age, gender, race, education level, smoking, drinking, economic levels, physical activity, blood pressure, triglyceride, total cholesterol (TC), estimated glomerular filtration rate (eGFR), lipid-lowering medications, and antihypertensive medications were included as covariates of no interest into the analyses to correct for error correlations. Among these, race is categorized into non-Hispanic white, non-Hispanic Black, Mexican American, and others. Education level was divided into three groups based on the completion of high school as the distinguishing criterion. Using self-reported data, tobacco smoking status was categorized into three groups: never smokers (smoked <100 cigarettes), former smokers (smoked≥100 cigarettes but had currently quit smoking), and current smokers. We categorized current drinkers according to their alcohol intake as mild drinkers, moderate drinkers, and heavy drinkers. The economic levels were quantified as the poverty-income ratio (PIR), which represents the household income relative to the federal poverty line. These levels were categorized into three groups based on two thresholds: 1.3 and 3.5. The respondents’ weekly activity level was evaluated using metabolic equivalents of task (METs). Additionally, eGFR was estimated utilizing the creatinine equation developed by the Chronic Kidney Disease Epidemiology Collaboration (CKD-EPI) in 2009 (19). All variables were collected simultaneously with prediabetes and diabetes prevalence.

### 2.4 Statistical analysis

The baseline characteristics of the participants were described by VAI quartiles. Continuous variables were expressed as means ± standard deviation and categorical variables as percentages. One-way ANOVA and chi-square tests were used to compare the differences between the four groups. To elucidate the association between VAI and prediabetes and diabetes, we constructed three multiple logistic regression models while adjusting for various covariates. Additionally, a generalized additive model (GAM) was employed to fit the dose-response curve. The characteristics of the fitted smooth curve guided the application of a two-part logistic regression model to investigate potential non-linear associations. A comparison was made between standard and segmented logistic regression models using the log-likelihood ratio test to identify any turning points (considered significant at P<0.05). Moreover, we investigated whether the relationship between VAI and prediabetes and diabetes varied across different subgroups stratified by baseline characteristics such as gender, age, smoking status, alcohol consumption, and hypertension. All data were processed and analyzed using R 3.5.3 and EmpowerStats software, and statistical significance was defined as P < 0.05.

## 3 Results

### 3.1 Baseline characteristics of participants

The study enrolled a cohort of 24,072 individuals with an average age of 47.30 ± 19.08 years, among whom 51.43% were female. **Table 1** displayed the baseline characteristics of the participants, described based on the quartile distribution of the VAI. Statistically significant differences were noted for all variables except METs/week among the four VAI groups. Compared with the group with lower VAI levels, participants in the highest VAI quartile (Q4) were characterized as older, more women, more non-Hispanic white, lower educational level, more current or former smokers, higher frequency of alcohol consumption, lower PIR, higher blood pressure levels, and lower eGFR. Most notably, the prevalence of prediabetes and diabetes was also higher in those with higher VAI levels.

**Table 1:** Baseline characteristics of the participants.

Characteristic	VAI quartiles				P-value
	Q1 (0.09-0.88) N=6018	Q2 (0.88-1.45) N=6018	Q3 (1.45-2.45) N=6018	Q4 (2.45-10.44) N=6018	
Age (years)	42.77 ± 19.29	46.51 ± 19.35	49.47 ± 18.86	50.45 ± 17.86	<0.001
Gender (%)					<0.001
Male	53.79	47.92	46.46	46.10	
Female	46.21	52.08	53.54	53.90	
Race/ethnicity (%)					<0.001
Non-Hispanic white	37.42	42.90	44.52	48.55	
Non-Hispanic Black	32.49	22.93	16.45	10.30	
Mexican American	12.99	16.93	20.99	24.13	
Others	17.10	17.23	18.05	17.02	
Educational level (%)					<0.001
Less than high school	22.91	25.89	29.63	33.63	
High school	22.65	24.13	23.71	24.58	
More than high school	54.44	49.98	46.66	41.80	
Smoking (%)					<0.001
Never	60.09	56.38	53.32	49.15	
Former	21.64	23.88	26.16	27.59	
Now	18.26	19.74	20.52	23.26	
Drinking (%)					<0.001
Never	13.24	14.27	14.31	16.59	
Former	11.95	15.49	19.37	21.96	
Mild	37.20	33.80	32.85	29.94	
Moderate	17.72	15.90	13.50	12.03	
Heavy	19.88	20.54	19.96	19.48	
PIR (%)					<0.001
Low	28.91	29.99	31.32	34.80	
Medium	37.54	37.57	39.72	38.39	
High	33.55	32.44	28.96	26.81	
METs/week (%)					0.097
Low	95.24	94.90	95.55	94.96	
Moderate	2.37	3.02	2.62	3.15	
Vigorous	2.39	2.09	1.83	1.89	
BMI (kg/m²)	25.61 ± 5.83	27.88 ± 6.37	29.83 ± 6.71	31.01 ± 6.34	<0.001
SBP (mmHg)	119.76 ± 17.98	121.79 ± 18.62	123.84 ± 19.13	125.93 ± 19.39	<0.001
DBP (mmHg)	67.99 ± 11.57	69.06 ± 11.92	69.69 ± 12.24	70.84 ± 12.48	<0.001
TG (mg/dl)	58.38 ± 17.18	89.74 ± 20.56	127.36 ± 29.44	221.50 ± 80.22	<0.001
TC (mg/dl)	179.72 ± 37.10	188.33 ± 39.24	195.78 ± 41.12	207.55 ± 44.55	<0.001
eGFR (ml/min/1.73 m^2^)	102.28 ± 23.58	97.60 ± 24.12	94.90 ± 24.61	93.22 ± 25.25	<0.001
Lipid-lowering medications (%)	11.06	14.87	19.03	21.49	<0.001
Antihypertensive medications (%)	19.02	25.41	31.73	36.51	<0.001
Glucose metabolism state (%)					<0.001
Normal	61.25	52.18	42.92	33.50	
Prediabetes	30.13	34.53	37.54	38.92	
Diabetes	8.62	13.29	19.54	27.58	

VAI, visceral adiposity index; PIR, poverty income ratio; MET, metabolic equivalent of task; SBP, systolic blood pressure; DBP, diastolic blood pressure; TG, triglyceride; TC, total cholesterol; eGFR, estimated glomerular filtration rate.

### 3.2 Relationship between VAI and prediabetes and diabetes

A multivariate logistic regression analysis was conducted to assess the correlation between VAI and prediabetes and diabetes, as presented in **Table 2**. The results consistently demonstrated positive associations between continuous VAI values and prediabetes and diabetes across all models, regardless of adjustment for confounding factors. In addition, participants were stratified into quartiles based on their VAI levels, using the first quartile (Q1) as the reference category. We found that with a quarter increase in VAI, there was a significant elevation in the ORs for prediabetes and diabetes, indicating a notable contribution of elevated VAI levels to the prevalence of these conditions (P-value for trend <0.001).

**Table 2:** Relationship between the VAI and prediabetes and diabetes in different models.

VAI	Model 1	Model 2	Model 3
Continuous	1.32 (1.29, 1.34)	1.30 (1.27, 1.32)	1.71 (1.59, 1.85)
Quartiles			
Q1(0.09-0.88)	Reference	Reference	Reference
Q2(0.88-1.45)	1.45 (1.35, 1.56)	1.37 (1.27, 1.49)	1.37 (1.23, 1.53)
Q3(1.45-2.45)	2.10 (1.95, 2.26)	1.89 (1.74, 2.05)	1.87 (1.65, 2.12)
Q4(2.45-10.44)	3.14 (2.91, 3.38)	2.93 (2.69, 3.19)	2.80 (2.33, 3.37)
P for trend	<0.001	<0.001	<0.001

Model 1: Non-adjusted.

Model 2: Adjusted for age, gender, race/ethnicity, and education level.

Model 3: Adjusted for age, gender, race/ethnicity, education level, smoking, drinking, PIR, METs/week, SBP, TG, TC, eGFR, lipid-lowering medications, and antihypertensive medications.

VAI, visceral adiposity index; PIR, poverty income ratio; MET, metabolic equivalent of task; SBP, systolic blood pressure; TG, triglyceride; TC, total cholesterol; eGFR, estimated glomerular filtration rate.

**Figure 2:**
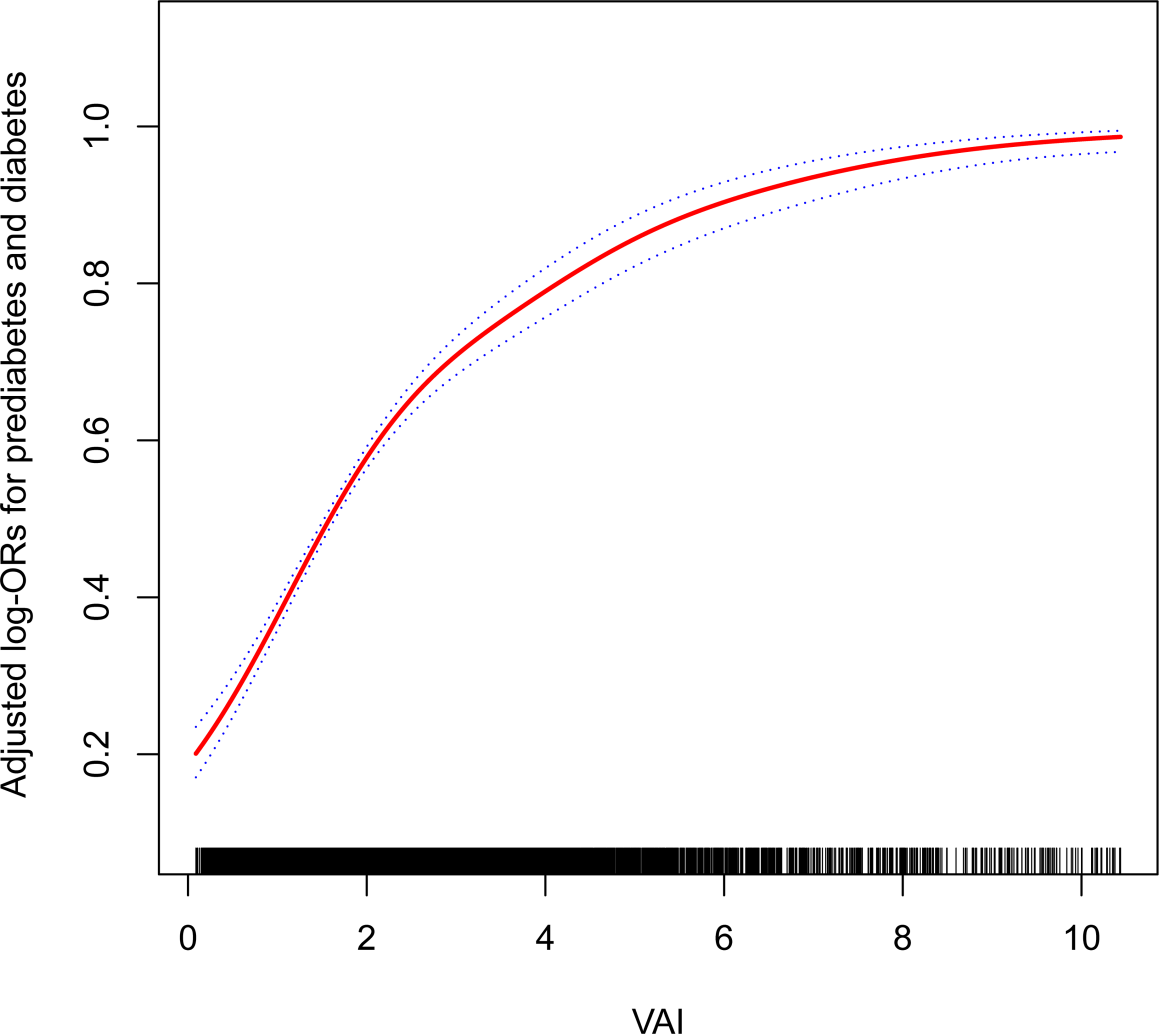
The non-linear association between the VAI and the prevalence of prediabetes and diabetes. Age, gender, race/ethnicity, education level, smoking, drinking, PIR, METs/week, SBP, TG, TC, eGFR, lipid-lowering medications, and antihypertensive medications were adjusted for. VAI, visceral adiposity index; PIR, poverty income ratio; MET, metabolic equivalent of task; SBP, systolic blood pressure; TG, triglyceride; TC, total cholesterol; eGFR, estimated glomerular filtration rate.

Furthermore, the results obtained by fitting a smooth curve and the two-part logistic regression model suggested that there is also a non-linear association between VAI and prediabetes and diabetes (**Table 3**, **Figure 2**). The inflection point was found to be 2.10 after the threshold effect analysis. When the VAI was <2.10, the OR (95% CI) was 2.47 (2.21,2.76); when the VAI> 2.10, the OR (95% CI) was 1.57 (1.46,1.70). This shows that before and after the inflection point, VAI was significantly positively correlated with prediabetes and diabetes. The log-likelihood ratio test showed statistical differences in the slopes between the standard logistic regression model and the piecewise logistic regression model (p<0.001).

**Table 3:** Threshold effect analysis of the VAI on prediabetes and diabetes using a two-part logistic regression model.

VAI	Adjusted OR^*^ (95% CI)	P-value
Model I		
Fitting by the standard linear model	1.71 (1.59, 1.85)	<0.0001
Model II		
Inflection point	2.10	
< Inflection point	2.47 (2.21, 2.76)	<0.0001
> Inflection point	1.57 (1.46, 1.70)	<0.0001
Log likelihood ratio	/	<0.001

^*^Adjusted for age, gender, race/ethnicity, education level, smoking, drinking, PIR, METs/week, SBP, TG, TC, eGFR, lipid-lowering medications, and antihypertensive medications.

VAI, visceral adiposity index; OR, odd ratio; CI, confidence interval.; PIR, poverty income ratio; MET, metabolic equivalent of task; SBP, systolic blood pressure; TG, triglyceride; TC, total cholesterol; eGFR, estimated glomerular filtration rate.

### 3.3 Subgroup analyses

**Figure 3:**
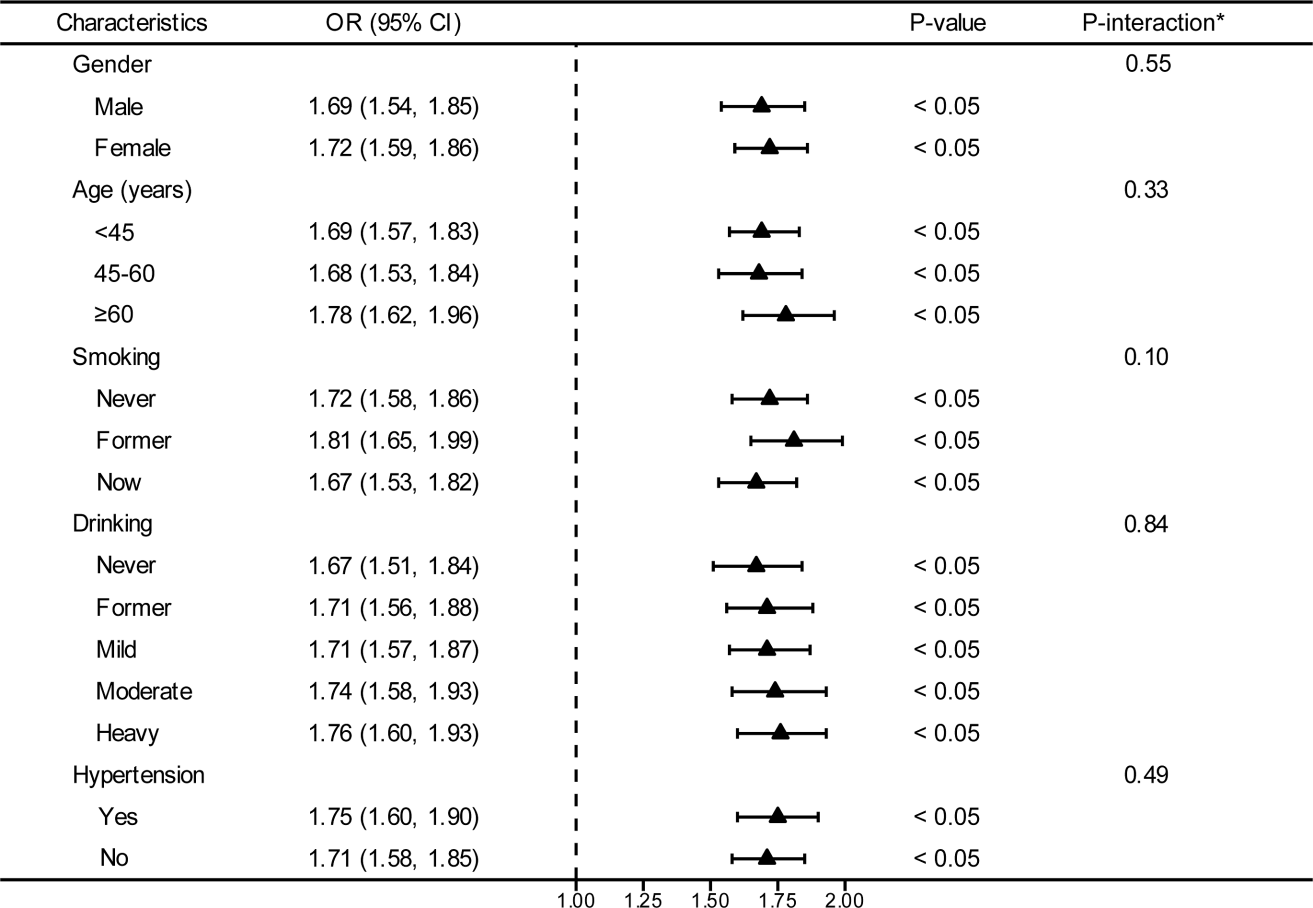
Stratified analyses between the VAI and the prevalence of prediabetes and diabetes. ^*^Each stratification adjusted for all the factors (age, gender, race/ethnicity, education level, smoking, drinking, PIR, METs/week, SBP, TG, TC, eGFR, lipid-lowering medications, and antihypertensive medications) except the stratification factor itself. OR, odd ratio; CI, confidence interval; VAI, visceral adiposity index; PIR, poverty income ratio; MET, metabolic equivalent of task; SBP, systolic blood pressure; TG, triglyceride; TC, total cholesterol; eGFR, estimated glomerular filtration rate.

**Figure 4:**
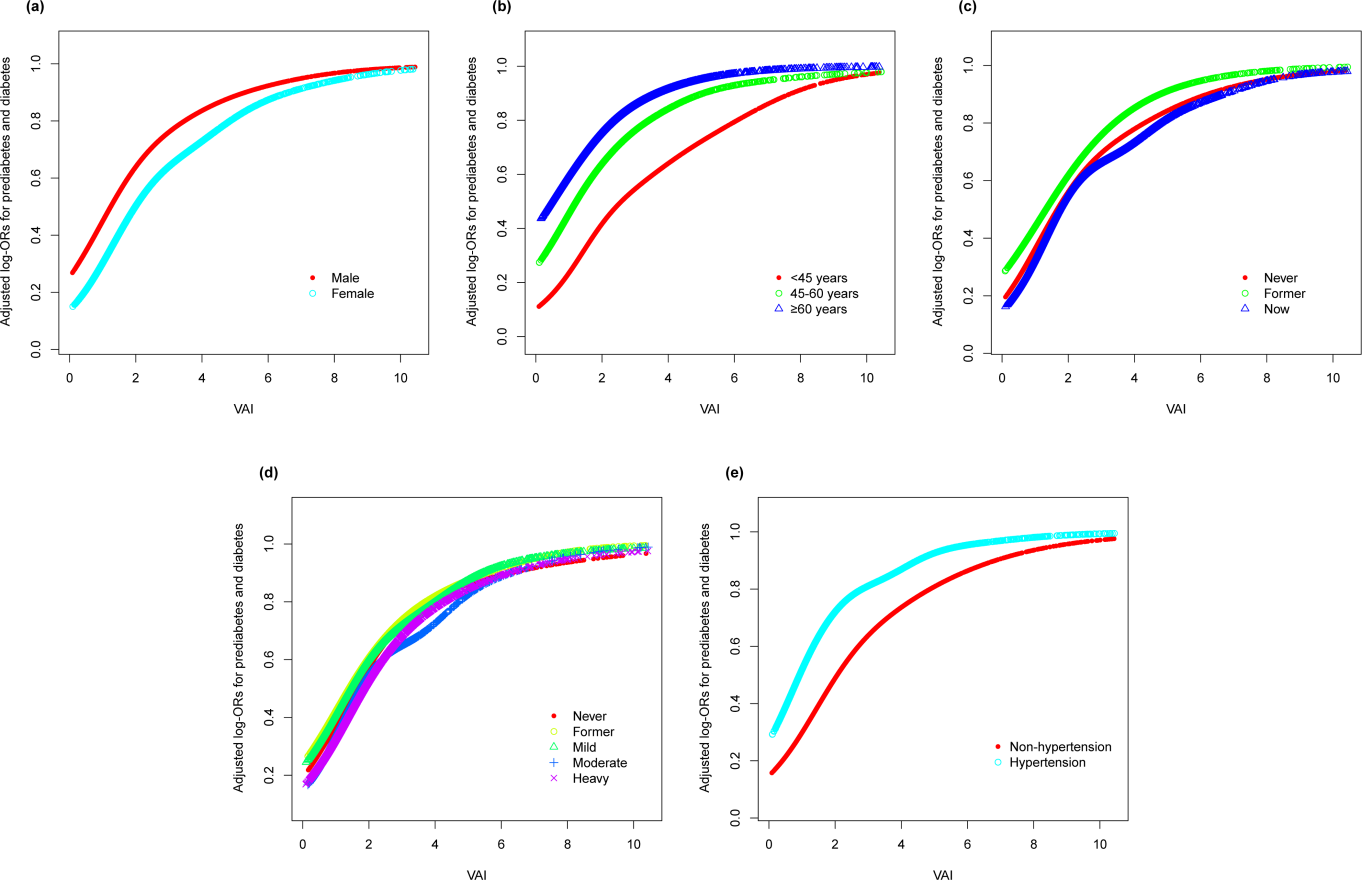
Stratified analyses (by **(a)** gender; **(b)** age; **(c)** smoking; **(d)** drinking; **(e)** hypertension) between VAI and the prevalence of prediabetes and diabetes using generalized additive model and smooth curve fittings. ^*^Each generalized additive model and smooth curve fitting was adjusted for all factors, including age, gender, race/ethnicity, education level, smoking, drinking, PIR, METs/week, SBP, TG, TC, eGFR, lipid lowering medications, and antihypertensive medications, except for the stratification factor itself. VAI, visceral adiposity index; PIR, poverty income ratio; MET, metabolic equivalent of task; SBP, systolic blood pressure; TG, triglyceride; TC, total cholesterol; eGFR, estimated glomerular filtration rate.

In an effort to ascertain the robustness of the association between VAI and prediabetes and diabetes, we executed stratified subgroup analyses alongside interaction testing (**Figure 3**). Our findings indicated a maintained positive correlation between VAI and both prediabetes and diabetes across various strata defined by gender, age, smoking habits, alcohol intake, and hypertension. In addition, no significant interaction was reported in all subgroups, indicating that the positive correlation between VAI and prediabetes and diabetes was not related to the above stratification parameters (all p>0.05 for interactions). The stratified analyses revealed that the non-linear correlations of each subgroup were consistent with the overall trend, and there was no significant heterogeneity among different subgroups (**Figure 4**).

## 4 Discussion

The purpose of this study was to assess the relationship between the VAI and prediabetes and diabetes among a sample of adult individuals sourced from NHANES data collected from 1999 to 2018. Our results demonstrated a notable positive association between the VAI and both prediabetes and diabetes, which persisted regardless of adjustments for confounding variables. In addition, we found a non-linear relationship between the VAI and both prediabetes and diabetes and determined an inflection point of 2.10 for the VAI level. When the population was stratified by gender, age, smoking, drinking, and hypertension, the results were consistent with the overall population, and no effect modifiers were detected that influenced the changes in the association of the VAI with prediabetes and diabetes. These findings supported the potential utility of VAI as a predictive tool for recognizing individuals susceptible to prediabetes and diabetes in an early stage.

The association of the VAI, as an index innovatively devised to gauge visceral adiposity function, with diabetes has been established in many previous studies (18, 20). A meta-analysis reported a significant positive correlation between the VAI and diabetes, which emphasizes the potential role of the VAI in the development of diabetes (18). Similarly, an aggregated examination of 216 longitudinal studies demonstrated that every unit increase in the VAI is associated with a 42% increase in the likelihood of developing diabetes (21). A study in the Chinese population showed that compared with TG, HDL-C, and other indicators, the VAI had obvious advantages in predicting diabetes in normoglycemic subjects (22).

There are a large number of people in the prediabetic state, and many patients may even be undetected. If not taken seriously, they may progress to diabetes and have an increased risk of developing many chronic diseases. Therefore, we included the prediabetic population in this study, together with diabetes as an outcome variable, to evaluate their association with the VAI. We found that subjects in the uppermost quarter of VAI had a 2.8 times increased likelihood of prediabetes and diabetes compared to those in the lowest quarter. A study of the Chinese population supports our findings (23). Similarly, a meta-analysis of 112,603 participants showed that VAI might increase the risk for prediabetes (24). In addition, in the German population study, VAI was also found to have high sensitivity for the identification of both prediabetes and diabetes, and its ability to distinguish abnormal blood glucose was comparable to that of HOMA-IR, an established marker for the diagnosis of insulin resistance (25). In addition, our subgroup analyses showed that the association of the VAI with prediabetes and diabetes was independent of factors such as age, gender, smoking, alcohol consumption, and hypertension. This suggests that the VAI may be a potential independent risk indicator for prediabetes and diabetes.

The presence of excessive abdominal fat is linked to an increased likelihood of insulin resistance and impaired β-cell function (26). The VAI, a proxy for cardiometabolic risk in healthy individuals, has shown a significant inverse correlation with insulin sensitivity (14). The biological pathways through which heightened VAI levels contribute to the augmented risk of prediabetes and diabetes potentially involve impacts on insulin resistance, pancreatic β-cell function, and adiponectin levels (27). Adipose tissue is known to release multiple pro-inflammatory factors and adipokines, fostering a chronic inflammatory state that can induce β-cell damage and exacerbate insulin resistance, eventually leading to diabetes (28, 29). Secondly, a high level of free fatty acids in individuals with obesity increases TG storage in the muscle and liver, reduces insulin sensitivity, and causes lipotoxic responses (30, 31). In addition, studies have shown that the VAI is the only determinant of adiponectin levels and can play an indirect role in impaired adiponectin levels and glucose metabolism (32, 33).

It is noteworthy that the relationship between the VAI and prediabetes and diabetes also exhibited a non-linear pattern. However, the results of previous studies are still controversial. A study was consistent with our results in patients with hypertension (34). Furthermore, a dose-response meta-analysis of longitudinal studies also revealed a monotonic positive association between the VAI and the risk of diabetes (21). In contrast, the study by Fang et al. stated that no non-linear relationship was detected between the VAI and diabetes (18). This disparity could stem from variations in participant selection criteria or might be ascribed to dissimilarities in the research methodologies and designs employed. In our study, we observed a significant non-linear relationship between the VAI and the prevalence of prediabetes and diabetes, which showed a parabolic curve trend. Specifically, the risk of prediabetes and diabetes increased significantly with increasing VAI values. However, after the VAI values exceeded a specific threshold of 2.10, the rate of increase plateaued, although the risk remained high. The explanations for these results may be as follows: first, excessive accumulation of visceral fat may adversely affect metabolic processes such as insulin sensitivity and inflammatory response, thereby increasing the risk of diabetes. However, when visceral fat accumulates to a certain extent, the increased risk may no longer follow a simple linear pattern due to limitations in biological mechanisms or individual metabolic differences. In addition, we need to consider possible biases in study design and data collection. A significant proportion of overweight individuals or individuals with obesity may have been excluded from the study due to death, serious illness, or other reasons that precluded participation in the interview. Nonetheless, the identification of the VAI inflection point provides a reference value for clinicians to assess an individual’s risk of developing prediabetes or diabetes more accurately. At the same time, this finding also highlights the need for further research on the VAI and its complex relationship with prediabetes and diabetes risk.

Nonetheless, the research has certain limitations. First, it should be noted that the study relies exclusively on a cross-sectional methodology, which ultimately obstructs the determination of a causal connection regarding the relationship between the VAI and prediabetes and diabetes. Consequently, further extensive prospective studies are required to validate these findings. Second, it is important to acknowledge that the dataset employed for this study originated from the NHANES database, which may restrict its generalizability across diverse ethnicities and populations. Moreover, a considerable number of participants who lacked essential data for VAI calculation were excluded from the analysis. Finally, despite our consideration of various potential effect modifiers, there remains a possibility of unidentified confounders leading to selection bias. Therefore, cautious interpretation is warranted when considering the outcomes derived from this investigation.

## 5 Conclusions

This cross-sectional study, utilizing the NHANES database, has substantiated a non-linear positive association between the VAI and prediabetes and diabetes. These findings suggest that the VAI has potential as a biomarker for predicting the onset of prediabetes and diabetes, offering novel perspectives for risk evaluation and preventive healthcare approaches. Nevertheless, further prospective cohort studies are warranted to validate these observations.

## Data Availability

Publicly available datasets were analyzed in this study. This data can be found here: https://www.cdc.gov/nchs/nhanes/index.htm.
